# Comparative, Controlled, Retrospective Study of Patient-Reported Outcomes After Meniscectomy With Adjunctive Use of Platelet-Rich Plasma or Amniotic Umbilical Cord Tissue

**DOI:** 10.31486/toj.23.0073

**Published:** 2024

**Authors:** Nneoma Duru, Gerard Williams, Eric Assid, Andrew Renshaw, Deryk Jones

**Affiliations:** ^1^ Ochsner Andrews Sports Medicine Institute, Ochsner Clinic Foundation, Jefferson, LA; ^2^ Department of Orthopedic Surgery, Howard University Hospital, Washington, DC; ^3^ Department of Internal Medicine, Medical College of Georgia, Augusta University, Augusta, GA; ^4^ The University of Queensland School of Medicine, Ochsner Clinical School, New Orleans, LA

**Keywords:** *Amnion*, *platelet-rich plasma*, *umbilical cord*

## Abstract

**Background:** Meniscal tears are one of the most frequent injuries to the knee, with an estimated incidence of 222 per 100,000 individuals aged 18 to 55 years based on magnetic resonance imaging. Poor outcomes following meniscal surgical interventions are common and have led many surgeons to use biologic augmentation strategies to enhance the healing.

**Methods:** We conducted a single-center, retrospective, observational study of patients who underwent arthroscopic meniscectomy with and without adjunctive platelet-rich plasma (PRP) or the particulate form of amniotic umbilical cord (AMUC) tissue. We evaluated patient-reported outcomes on the visual analog scale for pain, International Knee Documentation Committee (IKDC) Subjective Knee Evaluation Form, Lysholm Knee Scoring Scale, 12-Item Short Form Survey, and Knee Injury and Osteoarthritis Outcome Score (KOOS) during a 1-year postoperative period. Complications and follow-up procedures were also evaluated.

**Results:** We evaluated 113 patients who underwent meniscectomies from November 2010 to March 2017. Pain severity was significantly decreased only in the AMUC group at 6 months (*P*=0.0143). Patients in the AMUC group demonstrated significant improvement in functional recovery based on the IKDC and the KOOS subscales of pain, symptoms, activities of daily living, and sport and recreation function at 6 months. Patients in the PRP group had a significant benefit in the KOOS subscales of pain, symptoms, sport and recreation function, and knee-related quality of life at 3 months. Improvement in the control group was less substantial. Patients in the PRP group had more complications and follow-up procedures (30.0%) than patients in the AMUC group (8.3%).

**Conclusion:** In our study population, arthroscopic meniscectomy with adjunctive use of AMUC tissue improved patient-reported outcomes and reduced the reoperation rate compared to conventional technique or adjunctive use of PRP.

## INTRODUCTION

The meniscus is a crucial fibrocartilaginous component located between the femur and tibia on the periphery of the articular surfaces. Menisci are important for load distribution, knee stability, and prevention of articular cartilage degeneration,^1,2^ but the axial, rotational, and shear forces acting on the meniscus make it susceptible to injury.^1^ Meniscal lesions are the most commonly occurring intra-articular knee injury, with magnetic resonance imaging showing 222 tears per 100,000 individuals aged 18 to 55 years.^3^ Because of the importance of the meniscus to knee stability and function, the development of optimal methods to restore the tissue anatomically and functionally is warranted.

Patients with a torn meniscus usually experience knee pain, swelling, and a variety of mechanical symptoms, including popping and/or locking of the knee.^4^ Current methods of treating the injury most often involve arthroscopic partial meniscectomy, the most frequent surgical procedure performed by orthopedic surgeons with an estimated incidence in the United States of 17 per 100,000 individuals.^5,6^ Although widely practiced, arthroscopic partial meniscectomy has failed to show any substantial benefit compared to conservative treatment.^4,7,8^ At 1 year postoperatively, approximately 37% to 48% of patients are not satisfied with their knee function, and 59% to 65% consider the surgical treatment a failure.^8^ To ensure that function is maintained and long-term changes to other joints are avoided, preservation of the meniscus is essential.^4^

To overcome the inherent limitations in healing related to poor vascularity and heterogeneous cellularity, biologic augmentation techniques have been devised to enhance postoperative recovery after meniscectomy. Some of the injectable orthobiologics being explored for this indication include platelet-rich plasma (PRP) and amniotic umbilical cord (AMUC) tissue; both are composed of various growth factors, chemokines, and cytokines.^9,10^ Preclinical and clinical studies have shown that growth factors enhance the healing of meniscus repair by promoting chemotaxis, cellular proliferation, and matrix production at the repair site.^11^ Despite the use of PRP and AMUC tissue in meniscal repair and other orthopedic applications, outcomes after meniscectomies have not been compared between patients who received PRP vs AMUC tissue.^12,13^ In this retrospective study, we compared patient-reported outcomes of patients who underwent meniscectomy with and without the adjunctive use of PRP or AMUC tissue. We hypothesized that the use of the biologics would result in improved patient outcomes.

## METHODS

This study received approval from the institutional review board and was performed in accordance with the Declaration of Helsinki. We reviewed the medical records of patients who underwent meniscectomy performed by the principal investigator (DJ) at the Ochsner Sports Medicine Institute between November 2010 and March 2017. The PRP group (n=40) was treated from 2010 to 2013, the control group received no biologics (n=49) and underwent surgery from 2014 to 2016, and the AMUC group (n=24) underwent surgery from 2016 to 2017. Mitigating bias required excluding patients before we reviewed their outcomes to ensure our criteria were not influenced. All groups were held to the same exclusion criteria. Patients were excluded if they had extensive knee osteoarthritis, anterior cruciate ligament injury, varus/valgus knee realignment, or knee multiligament repairs, or if they did not have preoperative and postoperative outcome measurements. Demographic variables collected at baseline were sex; age; body mass index (BMI); and grade, size, and side of the injury. During the procedure, the cartilage was evaluated and graded according to the International Cartilage Repair Society (ICRS) classification system.^14^

Meniscectomy was performed using standard technique with a full radius 4.2 shaver (CONMED Corporation). For patients who received biologic augmentation, 10 cc of leukocyte-poor PRP (CASCADE Autologous Platelet System, MTF Biologics) or 100 mg of AMUC tissue (CLARIX FLO, Amniox Medical, Inc) in 2 cc of saline was injected intra-articularly into the knee joint using an 18-gauge spinal needle at the end of the procedure when incisions were closed. All persistent intra-articular fluid was aspirated using the same 18-gauge spinal needle prior to injection.

Postoperatively, all patients followed the same standardized rehabilitation protocol that started within the first week after the procedure under the supervision of a physiotherapist. The rehabilitation protocol focused on protected weight-bearing as tolerated and advancing to full-load weight-bearing over 4 to 6 weeks based on symptoms of pain, swelling, and range of motion. Low-impact exercise—exercycle and elliptical trainer usage—was allowed at 4 to 6 weeks. Full return to activities as tolerated was allowed at 6 to 8 weeks.

Patient-reported outcome measures were the visual analog scale (VAS) score for pain, International Knee Documentation Committee (IKDC) Subjective Knee Evaluation Form score, Lysholm Knee Scoring Scale score, 12-Item Short Form Survey (SF-12), and the Knee Injury and Osteoarthritis Outcome Score (KOOS). Each of the KOOS questionnaire subscales (pain, symptoms, activities of daily living, sport and recreation function, and knee-related quality of life) and the SF-12 components (physical component score [PCS] and mental component score [MCS]) were included for analysis. Higher scores correlate with improvement for all outcome measures except the VAS for which lower scores indicate improved outcomes. Outcome measure scores were collected preoperatively and in the immediate postoperative period (3 months, 6 months, and 12 months after surgery). The incidence of complications and reoperations was also assessed.

### Statistical Analysis

Descriptive statistics are used to characterize the study endpoints and are reported as mean ± SD. Patients who had missing intervals for patient-reported outcomes were investigated and determined to be eligible to remain in the analysis if follow-up interval outcomes were resumed (eg, the 3-month outcome scores were missing but the 6-month scores were available). No patients without preoperative outcome scores were included. Continuous outcome measures were evaluated using a *t* test. Descriptive statistics were performed for the entire sample on demographic characteristics (sex, age, and BMI), as well as grade, side, follow-up procedures, and infection/complication. Proc mixed repeated measures was used to test the effect of time on outcome measures—accounting for the effect of other variables to ensure that the conclusions drawn about other injection effects were unbiased—and the mean change between preoperative measures and postoperative times (eg, 3 months, 6 months, and 12 months). A *P* value <0.05 was considered statistically significant. A formal power analysis was not performed because of the retrospective nature of the study. All data were recorded using an Excel spreadsheet (Microsoft Corporation).

## RESULTS

A total of 113 patients met the inclusion criteria and were included in the study: 24 in the AMUC group (21.2%), 40 in the PRP group (35.4%), and 49 in the control group (43.4%). Preoperative and demographic data for the 3 treatment groups are presented in Table 1. Significant differences in baseline data between the groups were seen in sex, age, and ICRS grade (*P*<0.05). Post hoc analysis showed sex and ICRS grades were significantly different between the PRP and control groups, potentially because of the relatively high prevalence of male patients and ICRS grade 0 patients in the PRP group. The average age of the patients at the time of meniscectomy was 49.7 ± 13.9 years.

**Table 1.** Demographic Data of Study Population

Variable	AMUC Group, n=24	PRP Group, n=40	Control Group, n=49	*P* Value
Sex				**0.0379**
Male	13 (54.2)	29 (72.5)	22 (44.9)	
Female	11 (45.8)	11 (27.5)	27 (55.1)	
Age, years, mean ± SD	47.4 ± 15.3	47.0 ± 15.0	53.0 ± 11.8	**0.0259**
Body mass index, kg/m^2^, mean ± SD	31.9 ± 7.1	30.9 ± 6.7	31.1 ± 5.4	0.9385
ICRS grade				**0.0005**
0	5 (20.8)	21 (52.5)	10 (20.4)	
1	13 (54.2)	12 (30.0)	21 (42.9)	
2	6 (25.0)	7 (17.5)	18 (36.7)	
Side of tear				0.2350
Medial	16 (66.7)	29 (72.5)	26 (53.1)	
Lateral	6 (25.0)	8 (20.0)	13 (26.5)	
Medial and lateral	2 (8.3)	3 (7.5)	10 (20.4)	
Meniscus removed, %, mean ± SD	44.6 ± 22.3	51.5 ± 17.8	44.4 ± 25.7	

Note: Data are presented as n (%) unless otherwise indicated.

AMUC, amniotic umbilical cord tissue; ICRS, International Cartilage Repair Society; PRP, platelet-rich plasma.

### Visual Analog Scale

**Figure 1.**
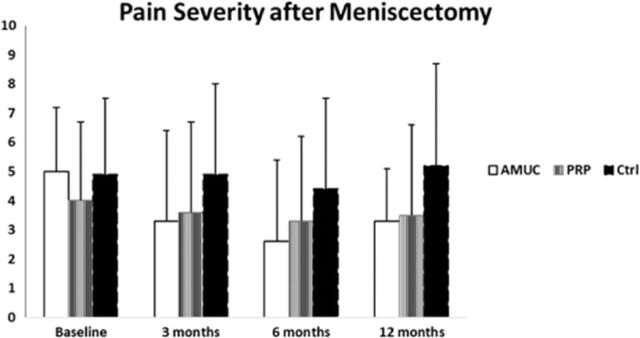
**Preoperative and postoperative visual analog scale pain severity scores by treatment group. Lower scores indicate better outcomes (decreased pain severity).** AMUC, amniotic umbilical cord tissue; Ctrl, control; PRP, platelet-rich plasma.

The average VAS pain severity significantly decreased only in the AMUC group at 6 months compared to baseline (*P*=0.0143) (Table 2, Figure 1). The reduction in pain severity scores was most notable in the AMUC group which demonstrated a reduction of 1.7 in pain at 12 months, compared to a reduction of 0.45 in the PRP group and an increase of 0.32 in the control group. Pain frequency was also significantly decreased in the AMUC group, declining from 8.13 at baseline to 4.33 at 3 months (*P*=0.0002) and to 4.00 at 6 months (not significant). In comparison, pain frequency scores in the control group were 5.00 at baseline, 5.20 at 3 months, 3.40 at 6 months, and 7.71 at 12 months (*P*>0.05 at each time point). Pain frequency scores in the PRP group increased from 3.65 at baseline to 6.00 at 6 months.

**Table 2.** Preoperative and Postoperative Visual Analog Scale Pain Scores

	Treatment Group					
	AMUC Group		PRP Group		Control Group	
Time Point	Frequency	Severity	Frequency	Severity	Frequency	Severity
Baseline	8.13 ± 2.24	5.00 ± 2.87	3.65 ± 2.59	4.00 ± 2.73	5.00 ± 2.11	4.86 ± 2.68
3 months	4.33 ± 2.79	3.33 ± 3.14	Low n	3.61 ± 3.14	5.20 ± 3.43	4.86 ± 3.08
	**0.0002**	0.0615		0.5680	0.8657	1.0000
6 months	4.00 ± 4.29	2.63 ± 2.83	Low n	3.28 ± 2.92	3.40 ± 3.37	4.38 ± 3.13
	0.0649	**0.0143**		0.3004	0.1869	0.4434
12 months	Low n	3.30 ± 1.79	Low n	3.55 ± 3.17	7.71 ± 3.50	5.18 ± 3.49
		0.1207		0.6714	0.0895	0.7764

Notes: Data are presented as mean ± SD. The term *Low n* indicates inadequate sample size. The visual analog scale is scored from 0 to 10, with 0 indicative of no pain and 10 indicative of the most severe pain. *P* values reflect the statistical significance of the change from baseline.

AMUC, amniotic umbilical cord tissue; PRP, platelet-rich plasma.

### Lysholm Knee Scoring Scale

**Figure 2.**
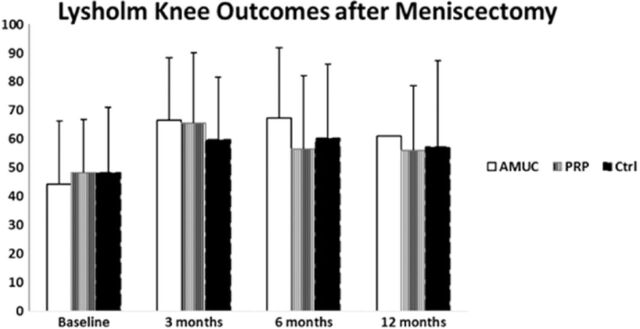
**Preoperative and postoperative Lysholm Knee Scoring Scale scores by treatment group. Higher scores indicate better outcomes.** AMUC, amniotic umbilical cord tissue; Ctrl, control; PRP, platelet-rich plasma.

Preoperatively, the mean Lysholm scores were 44.26 in the AMUC group, 48.41 in the PRP group, and 48.35 in the control group (Table 3, Figure 2). At 3 months postoperatively, the scores significantly improved to 66.47 (*P*=0.0046), 65.58 (*P*=0.0117), and 59.70 (*P*=0.0189) in the 3 groups, respectively. At 6 months postoperatively, the mean Lysholm score improved slightly but not significantly in the AMUC group (67.43) and the control group (60.24) but declined in the PRP group (56.64).

**Table 3.** Preoperative and Postoperative Lysholm Knee Scoring Scale and International Knee Documentation Committee (IKDC) Subjective Knee Evaluation Form Scores

	Treatment Group					
	AMUC Group		PRP Group		Control Group	
Time Point	Lysholm	IKDC	Lysholm	IKDC	Lysholm	IKDC
Baseline	44.26 ± 21.98	36.43 ± 17.06	48.41 ± 14.38	40.26 ± 2.73	48.35 ± 22.73	41.07 ± 19.99
3 months	66.47 ± 21.79	59.61 ± 16.91	65.58 ± 24.63	56.38 ± 20.76	59.70 ± 22.02	48.18 ± 21.07
	**0.0046**	**0.0003**	**0.0117**	**0.0053**	**0.0189**	0.1127
6 months	67.43 ± 24.45	60.09 ± 22.44	56.64 ± 25.50	47.85 ± 20.79	60.24 ± 25.90	52.17 ± 26.23
	0.0508	**0.0322**	0.3355	0.2772	0.0482	0.0610
12 months	Low n	Low n	56.00 ± 22.52	44.06 ± 16.11	57.36 ± 29.92	50.53 ± 28.53
			0.6216	0.7263	0.3669	0.3184

Notes: Data are presented as mean ± SD. The term *Low n* indicates inadequate sample size. For the Lysholm Knee Scoring Scale and the International Knee Documentation Committee (IKDC) Subjective Knee Evaluation Form, higher scores indicate better outcomes. *P* values reflect the statistical significance of the change from baseline.

AMUC, amniotic umbilical cord tissue; PRP, platelet-rich plasma.

### International Knee Documentation Committee Subjective Knee Evaluation Form

The IKDC scores demonstrated a trend similar to the Lysholm scores (Table 3). Compared to baseline scores, a significant increase in IKDC scores was seen at 3 and at 6 months in the AMUC group and at 3 months in the PRP group. The IKDC improved to 60.09 in the AMUC group, 47.85 in the PRP group, and 52.17 in the control group at 6 months, but only the AMUC group difference from baseline was significant.

### Knee Injury and Osteoarthritis Outcome Score

All 5 KOOS subscale scores showed significant improvement in the AMUC group at 3 months compared to baseline (Table 4), and 4 of the 5 KOOS subscale scores were significant at 6 months in the AMUC group. The AMUC group reached a relative plateau in all subscales at 6 months except for knee-related quality of life which markedly declined at 12 months. At the 3-month time point, 4 of the 5 KOOS subscale scores were significantly improved from baseline in the PRP group, while 2 of 5 KOOS subscale scores were significant in the control group at 3 months. Overall, the AMUC group showed better improvement in the KOOS compared to the PRP and control groups.

**Table 4.** Preoperative and Postoperative Knee Injury and Osteoarthritis Outcome Subscale Scores: Pain, Symptoms, Activities of Daily Living (ADL), Sport and Recreation Function (Sports), and Knee-Related Quality of Life (QOL)

	Treatment Group														
	AMUC Group					PRP Group					Control Group				
Time Point	Pain	Symptoms	ADL	Sports	QOL	Pain	Symptoms	ADL	Sports	QOL	Pain	Symptoms	ADL	Sports	QOL
Baseline	46.0 ± 23.2	49.8 ± 16.9	51.7 ± 25.5	31.3 ± 25.1	27.4 ± 20.6	51.4 ± 17.6	49.4 ± 21.1	61.6 ± 24.7	46.0 ± 26.6	25.8 ± 16.2	53.0 ± 20.2	60.1 ± 19.3	59.0 ± 22.6	44.8 ± 31.3	31.3 ± 25.3
3 months	70.9 ± 18.3	69.3 ± 16.0	78.8 ± 18.3	58.2 ± 26.9	47.1 ± 26.0	75.3 ± 22.1	71.2 ± 23.8	79.7 ± 25.9	68.9 ± 27.0	49.5 ± 30.1	66.6 ± 19.2	69.2 ± 18.8	68.5 ± 23.1	46.0 ± 33.1	42.1 ± 25.2
	**0.0008**	**0.0011**	**0.0005**	**0.0055**	**0.0208**	**0.0044**	**0.0156**	0.0802	**0.0410**	**0.0355**	**0.0017**	**0.0289**	0.0554	0.8626	0.0469
6 months	77.4 ± 15.7	74.5 ± 21.4	81.3 ± 13.9	57.1 ± 25.5	51.8 ± 31.2	68.9 ± 25.5	67.5 ± 25.8	73.1 ± 20.9	45.0 ± 38.6	22.9 ± 17.1	65.6 ± 24.4	69.2 ± 19.3	69.7 ± 24.9	53.2 ± 35.0	43.8 ± 31.3
	**0.0010**	**0.0219**	**0.0008**	**0.0405**	0.0899	0.0814	0.0860	0.2663	0.9513	0.7569	**0.0242**	0.0533	0.0665	0.3085	0.0763
12 months	Low n	Low n	Low n	Low n	Low n	55.6 ± 7.3	51.2 ± 7.4	55.4 ± 16.4	60.0 ± 13.2	33.3 ± 20.1	66.9 ± 27.6	69.3 ± 21.8	69.3 ± 27.6	56.5 ± 30.4	53.1 ± 28.1
						0.5668	0.8154	0.6329	0.2560	0.5998	0.1403	0.2449	0.2952	0.2941	**0.0264**

Notes: Data are presented as mean ± SD. The term *Low n* indicates inadequate sample size. For the Knee Injury and Osteoarthritis Outcome subscale scores, higher scores indicate better outcomes. *P* values reflect the statistical significance of the change from baseline.

AMUC, amniotic umbilical cord tissue; PRP, platelet-rich plasma.

### 12-Item Short Form Survey

The SF-12 PCS significantly improved only in the AMUC group (*P*=0.0103 at the 6-month time point), and the MCS significantly improved in the PRP and control groups at the 12-month time point (Table 5). The PCS improved by 13.12 from baseline in the AMUC group and by 0.07 in the PRP group at 12 months but decreased by 9.03 in the control group. The MCS improved by 13.15 in the control group at 12 months compared to baseline but decreased by 6.50 and 8.25 in the AMUC and PRP groups, respectively.

**Table 5.** Preoperative and Postoperative 12-Item Short Form Survey Physical Component Score (PCS) and Mental Component Score (MCS)

	Treatment Group					
	AMUC Group		PRP Group		Control Group	
Time Point	PCS	MCS	PCS	MCS	PCS	MCS
Baseline	34.58 ± 8.98	49.10 ± 12.40	51.82 ± 9.92	37.28 ± 8.70	51.36 ± 10.43	35.33 ± 10.20
3 months	40.75 ± 10.09	51.57 ± 10.60	53.98 ± 10.74	40.32 ± 10.99	51.41 ± 11.95	37.67 ± 12.80
	0.0668	0.5216	0.4792	0.3133	0.9833	0.3534
6 months	47.44 ± 9.45	52.73 ± 15.13	52.81 ± 8.78	37.82 ± 10.50	48.30 ± 11.65	42.90 ± 14.01
	**0.0103**	0.5796	0.7459	0.8733	0.2623	**0.0170**
12 months	47.70 ± Low n	42.60 ± Low n	51.89 ± 17.05	29.03 ± 2.06	42.33 ± 14.90	48.48 ± 11.57
			0.9965	**0.0179**	0.0960	**0.0059**

Notes: Data are presented as mean ± SD. The term *Low n* indicates inadequate sample size. For the 12-Item Short Form Survey physical component score (PCS) and mental component score (MCS), higher scores indicate better outcomes. *P* values reflect the statistical significance of the change from baseline.

AMUC, amniotic umbilical cord tissue; PRP, platelet-rich plasma.

### Complications and Follow-up Procedures

The rate of follow-up procedures was 8.3% in the AMUC group, 30.0% in the PRP group, and 40.8% in the control group (Table 6). The AMUC group demonstrated a numerically lower percent decrease in reoperations compared to the PRP and control groups, while a numerically lower percent decrease in reoperations was seen in the PRP group compared to the control group.

**Table 6.** Ipsilateral Complications and Follow-up Procedures

Complication/Procedure	AMUC Group, n=24	PRP Group, n=40	Control Group, n=49
Revision meniscectomy	1 (4.2)	5 (12.5)	7 (14.3)
Unicompartmental knee arthroplasty	0	2 (5.0)	2 (4.1)
Total knee arthroplasty	1 (4.2)	2 (5.0)	7 (14.3)
Bony realignment	0	1 (2.5)	1 (2.0)
Repair or transplant	0	0	3 (6.1)
Incision and drainage	0	2 (5.0)	0
Total	2 (8.3)	12 (30.0)	20 (40.8)

Note: Data are presented as n (%).

AMUC, amniotic umbilical cord tissue; PRP, platelet-rich plasma.

## DISCUSSION

Most orthopedic surgeons would agree on several goals during the immediate postoperative period following arthroscopic meniscectomy: controlling pain and swelling, maximizing knee range of motion, facilitating return to preinjury activities of daily living, and allowing functional muscle recovery. Short-term outcomes after meniscectomy are generally good, but systematic reviews and meta-analyses have found arthroscopic surgical intervention to provide no better outcome than placebo surgery or conservative management (focus on strength, flexibility, and functionality).^15-21^ Presumably based on these findings, orthopedic surgeons have begun to incorporate biologic augmentation techniques to enhance the healing process and increase the likelihood of better outcomes after meniscectomy.

The objective of this study was to compare the outcomes of these various techniques. Our results showed that biologic augmentation with PRP or AMUC tissue promoted better KOOS subscale outcomes at 3 months postoperatively compared to standard intervention (Table 4). Notable improvements in KOOS pain, symptoms, activities of daily living, and sport and recreation function were also evident at 6 months in the AMUC group.

The menisci are known to have poor healing potential because of limited vascularity in the central and middle third of the tissue. The potential value of using PRP and AMUC tissue in meniscectomy stems from their ability to deliver a high concentration of growth factors and cytokines to the injury site and augment healing in this limited environment. PRP contains transforming growth factor beta-1, platelet-derived growth factor, and vascular endothelial growth factor, all of which are involved in varying levels in cell chemotaxis, differentiation, and extracellular matrix production. Animal studies have confirmed these beneficial effects,^22^ but clinical studies have shown contradictory findings. Pujol et al^9^ found that clinical outcomes (KOOS subscales of pain and sport and recreation function) were slightly improved 2.7 years after open meniscal repair with PRP, whereas Griffin et al^23^ showed that IKDC and Lysholm outcomes were similar 4 years after arthroscopic meniscus repair with and without PRP. Our results show that PRP does not appreciably affect outcome values and are consistent with Griffin et al, perhaps suggesting a limited role of PRP in arthroscopic surgery. Reoperation rates in the 2 studies are also comparable: Griffin et al^23^ reported a reoperation rate of 27% in the PRP group and 25% in the control group, compared to 30.0% in our PRP group and 40.8% in our control group. Based on these findings, the clinical benefit of PRP for meniscectomy remains unclear.

The primary finding from this study was the rapid and significant improvement in outcomes in the AMUC group. Total knee arthroplasty was also notably decreased in the AMUC and PRP groups, with 4.2%, 5.0%, and 14.3% total knee arthroplasty cases in the AMUC, PRP, and control groups, respectively. Our control group results are consistent with prior studies showing that 14.3% to 18.8% of patients undergo total knee arthroplasty within 1 year after arthroscopic meniscectomy.^24,25^ AMUC tissue use may be advantageous in these cases because of its compositional components that modulate inflammation and promote healing.^26^ Placental tissues are processed to retain key biologic and structural components, including many growth factors, cytokines, and proteins such as HC-HA/PTX3 that promote regenerative healing.^10,26,27^ Placental tissue contrasts with PRP, a biologic that is theoretically more inclined to incite a proinflammatory environment with growth factors at supraphysiologic levels and may lead to poorer long-term outcomes, as seen in our study.

Short-term patient-reported outcomes after arthroscopic partial meniscectomy are not widely reported in the literature.^8^ In our study, patients in the AMUC group showed a maximum improvement of 31.4 points in the KOOS pain subscale at 6 months compared to baseline. This improvement was greater than the PRP group maximum improvement of 23.9 points in the KOOS pain subscale at 3 months and the control group maximum of 21.8 points in KOOS knee-related quality of life subscale at 12 months compared to baseline (Table 4). In contrast with prior studies,^8,28^ outcomes at 6 months and 12 months were relatively constant or slightly worse. This result is likely attributable to our postoperative protocol that does not require patients to return after 6 months; hence, patients returning at 12 months were generally in more pain. In addition, the older age in our cohort is often associated with worse outcomes and may have led to reduced scores at 12 months.^8^

Future prospective, controlled studies with longer-term follow-up are required to confirm the use of biologics in meniscal surgery. Meniscectomy is known to affect the biomechanical function of the knee by increasing the mean and peak contact stress which usually leads to the development of progressive osteoarthritis.^4^ Therefore, AMUC tissue may not only provide symptomatic relief in the short term but may also slow or prevent this progressive development of osteoarthritis and maintain knee function in the long term.

Limitations of our study include lack of generalizability because of the single-center analysis. Our study also included more males than females, further limiting our scope of generalizability. Patient questionnaires are a subjective measure of outcomes; the use of patient questionnaires may introduce a bias for patients who cannot maintain focus, comprehend the questionnaire reading level, or see the lettering properly. Additionally, the low number of patients at our 12-month follow-up represents gaps in data, so strong conclusions about longer term outcomes cannot be made. Retrospective analysis has inherent selection limitations despite efforts to adhere as closely as possible to a selection protocol. Patient compliance and motivation to complete the rehabilitation protocol are also limitations that can directly affect outcomes.

## CONCLUSION

In our study population, arthroscopic meniscectomy with adjunctive use of AMUC tissue improved patient-reported outcomes and reduced the reoperation rate compared to conventional surgery or adjunctive use of PRP.

## Notes

*This article meets the Accreditation Council for Graduate Medical Education and the American Board of Medical Specialties Maintenance of Certification competencies for Patient Care and Medical Knowledge.*
